# Drivers of Vertical HIV Transmission in Sub‐Saharan Africa and the Impact and Cost‐Effectiveness of Targeted and Universal Lenacapavir Pre‐Exposure Prophylaxis

**DOI:** 10.1002/jia2.70127

**Published:** 2026-06-19

**Authors:** Anna Yakusik, Magdalene K. Walters, Deepak Mattur, Savvy Brar, Leigh F. Johnson, Gesine Meyer‐Rath, Lise Jamieson, Angela Mushavi, John Stover, Mary Mahy

**Affiliations:** ^1^ Data and Evidence Department Joint United Nations Programme on HIV/AIDS Geneva Switzerland; ^2^ MRC Centre for Global Infectious Disease Analysis, School of Public Health Imperial College London London UK; ^3^ Sustainability Department Joint United Nations Programme on HIV/AIDS Geneva Switzerland; ^4^ Division of Data Analytics, Planning and Monitoring UNICEF New York City New York USA; ^5^ Centre for Integrated Data and Epidemiological Research School of Public Health University of Cape Town Cape Town South Africa; ^6^ Health Economics and Epidemiology Research Office Faculty of Health Sciences University of the Witwatersrand Johannesburg South Africa; ^7^ The South African Department of Science and Innovation/National Research Foundation Centre of Excellence in Epidemiological Modelling and Analysis (SACEMA) Stellenbosch South Africa; ^8^ Department of Global Health Boston University School of Public Health Boston Massachusetts USA; ^9^ Health Economics and Epidemiology Research Office Wits Health Consortium University of the Witwatersrand Johannesburg South Africa; ^10^ SACEMA Stellenbosch University Stellenbosch South Africa; ^11^ Ministry of Health and Child Care Harare Zimbabwe; ^12^ Center for Modelling Planning, and Policy Analysis Avenir Health Glastonbury Connecticut USA

**Keywords:** cost‐effectiveness analysis, HIV prevention, lenacapavir, maternal HIV acquisition, mathematical modelling, pre‐exposure prophylaxis, sub‐Saharan Africa, vertical HIV transmission

## Abstract

**Introduction:**

Eliminating vertical HIV transmission remains a major public health priority, particularly in sub‐Saharan Africa (SSA), which accounted for 83% of global paediatric HIV acquistions in 2024. Despite expanded antiretroviral therapy (ART) coverage, gaps in maternal ART access, retention and HIV acquisition during pregnancy and breastfeeding continue to drive paediatric HIV acquisitions. Long‐acting injectable (LAI) lenacapavir pre‐exposure prophylaxis (PrEP) may reduce paediatric HIV acquisitions by preventing maternal HIV acquisition. We evaluated drivers of vertical transmission in SSA and assessed the impact and cost‐effectiveness of LAI lenacapavir PrEP among pregnant and breastfeeding women (PBW) without HIV.

**Methods:**

Using 2025 UNAIDS estimates, Spectrum AIM and Naomi model outputs, we decomposed vertical HIV transmission pathways by maternal HIV acquisition timing and ART status. We modelled universal and geographically targeted rollout strategies using district‐level HIV incidence thresholds among women aged 15–49 years (≥0.7%, ≥0.5% and ≥0.3%). Base‐case assumptions included 65% uptake, 70% retention over 2.2 years, drug costs of US$40 per person‐year plus a US$17 loading dose and service delivery costs of US$50 per person‐year. Upper‐bound scenarios and deterministic sensitivity analyses evaluated implementation uncertainty.

**Results:**

In 2024, an estimated 98,000 new paediatric HIV acquisitions occurred in SSA. Lack of maternal ART access accounted for 46% of vertical transmissions, while ART discontinuation during pregnancy or breastfeeding contributed 19%. Maternal HIV acquisition during pregnancy or breastfeeding accounted for 25% of paediatric HIV acquisitions, reaching 59% in South Africa and 46% in Zambia. Under base‐case assumptions, universal LAI lenacapavir PrEP rollout averted approximately 56,100 HIV acquisitions at a net cost of US$85,200 per HIV acqusition averted. Geographic targeting at ≥0.7% incidence was more cost‐effective, averting approximately 8450 acquisitions at a net cost of US$8530 per acquisition averted. Retention and service delivery costs were the primary determinants of cost‐effectiveness.

**Conclusions:**

Gaps in maternal ART access and retention remain the dominant drivers of vertical HIV transmission in SSA, while maternal HIV acquisition contributes substantially in high‐incidence settings. Targeted LAI lenacapavir PrEP rollout among PBW without HIV could reduce maternal and paediatric HIV acquistions more efficiently than universal rollout, although outcomes remain highly sensitive to implementation conditions. LAI lenacapavir PrEP should complement strengthened maternal ART programmes, not replace them.

## 1 Introduction

Eliminating vertical transmission of human immunodeficiency virus (HIV) remains a critical global public health priority [1]. This challenge is particularly pronounced in sub‐Saharan Africa (SSA), where approximately 1.1 million women required antiretroviral therapy (ART) in 2024 to prevent vertical HIV transmission, accounting for 90% of the global total. Despite substantial efforts to scale up access to essential health services across the maternal−child HIV care continuum, only 86% of these women received ART [2]. This shortfall has contributed to an estimated 98,000 new paediatric HIV acquisitions in the region, representing 83% of the global burden [2].

Significant progress has been made over the past two decades [1, 2]. As a result, new paediatric HIV acquistions have declined by 65% since 2010, contributing to approximately 3.5 million acquisitions averted and substantial improvements in child survival [1, 2]. AIDS‐related mortality among children aged 0–14 years has also declined by approximately 71% [1, 2]. Evidence shows that effective viral suppression can reduce vertical transmission risk from 30% to 45% in untreated cases to below 1% with sustained ART [3, 4, 5, 6, 7]. The World Health Organization (WHO) “Treat All” policy [5], particularly Option B+ and rapid ART initiation, has been central to these gains.

Despite these achievements, important gaps remain. ART coverage and treatment outcomes among pregnant and breastfeeding women (PBW) living with HIV vary substantially across settings, with higher coverage generally observed in Eastern and Southern Africa [1, 2]. However, the relative contribution of different pathways to ongoing vertical HIV transmission remains insufficiently synthesized across settings.

New paediatric HIV acquisitions continue to occur due to a combination of factors [8, 9, 10, 11, 12, 13, 14]. Recent funding shortfalls further threaten progress towards elimination targets [2, 15, 16]. Long‐acting injectable (LAI) pre‐exposure prophylaxis (PrEP), including twice‐yearly lenacapavir, represents a novel prevention option for individuals who are HIV negative. Lenacapavir has demonstrated high efficacy in clinical trials [17, 18] and was recommended by WHO in 2025 as an additional PrEP option [19]. Its potential relevance for PBW lies in its ability to reduce maternal HIV acquisition during periods of elevated risk, thereby addressing one pathway contributing to vertical transmission of HIV. However, its population‐level impact at scale and cost‐effectiveness remain uncertain. Lenacapavir is administered as a subcutaneous injection every 6 months, offering a long‐acting alternative to daily oral PrEP and reducing reliance on continuous adherence during pregnancy and breastfeeding [17, 18, 19].

This study aims to disaggregate the current drivers of vertical HIV transmission in SSA and evaluate the impact and cost‐effectiveness of scaling up lenacapavir PrEP to reduce maternal HIV acquisition and associated paediatric HIV acquisitions.

Our specific objectives were to: (i) synthesize the drivers of vertical HIV transmission in SSA by timing of maternal HIV acquisition and ART status; and (ii) evaluate the impact and cost‐effectiveness of universal and geographically targeted lenacapavir PrEP rollout among HIV‐negative PBW under varying implementation assumptions using mathematical modelling and sensitivity analyses.

## 2 Methods

This study used a hierarchical analytical framework combining epidemiological decomposition and intervention modelling to evaluate vertical HIV transmission pathways and the projected impact of lenacapavir PrEP among HIV‐negative PBW in SSA.

We utilized 2025 Joint United Nations Programme on HIV/AIDS (UNAIDS) epidemiological estimates [1, 2, 20], incorporating surveillance, programme and modelling outputs through 2024 from the Spectrum AIDS Impact Module (AIM) [21] and the Naomi model [22]. These estimates provided national and subnational HIV burden measures used to characterize HIV burden globally and within SSA.

Analyses were restricted to SSA and stratified into Eastern and Southern Africa and Western and Central Africa to assess heterogeneity. Within each subregion, Spectrum AIM estimates were examined according to maternal HIV status, ART engagement and timing of maternal HIV acquisition.

Spectrum AIM is a deterministic compartmental model simulating maternal HIV progression, fertility and HIV transmission dynamics over time [21]. Maternal HIV status transitions between HIV negative, recent HIV acquisition, and established HIV states, with corresponding changes in transmission risk. Vertical transmission probabilities are conditional on maternal ART status, duration on ART and timing of HIV acquisition [23, 24].

Women receiving sustained ART are assigned regimen‐specific transmission probabilities stratified by immunological status, whereas women not receiving ART or discontinuing treatment are assigned higher transmission probabilities [23, 24, 25, 26]. The model incorporates ART initiation, retention and discontinuation, including increased transmission risk following treatment interruption, reflecting a rapid viral rebound [23, 24, 25, 26]. These transitions are integrated with fertility patterns, breastfeeding duration and time‐varying HIV acquisition risk to estimate stage‐specific contributions to vertical transmission.

**FIGURE 1:**
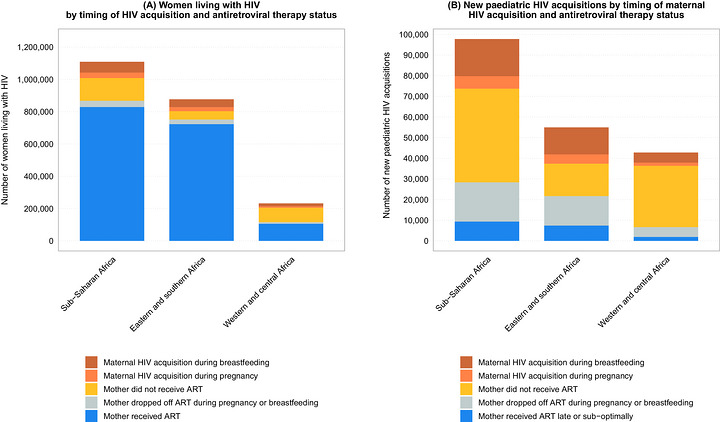
Drivers of vertical HIV transmission in sub‐Saharan Africa, 2024. Panel A shows women living with HIV stratified by timing of maternal HIV acquisition and antiretroviral therapy status. Panel B shows new paediatric HIV acquisitions stratified by the same categories. The stacked structure separates women living with HIV prior to pregnancy (lower three components, by ART status) from women who acquired HIV during pregnancy or breastfeeding (upper two components). Abbreviation: ART, antiretroviral therapy. *Source*: UNAIDS epidemiological estimates, 2025.

**FIGURE 2:**
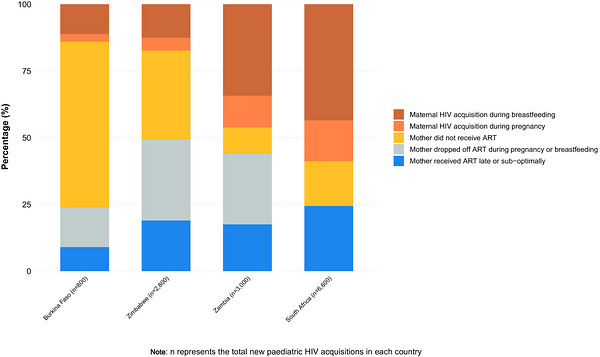
Percentage distribution of new paediatric HIV acquisitions by timing of maternal HIV acquisition and ART status in selected sub‐Saharan African countries, 2024. The stacked structure follows Figure 1, separating HIV acquisitions among infants born to women living with HIV prior to pregnancy (lower three components, by ART status) and HIV acquisitions associated with maternal HIV acquisition during pregnancy or breastfeeding (upper two components). Abbreviation: ART, antiretroviral therapy. *Source*: UNAIDS epidemiological estimates, 2025.

This framework enabled decomposition of paediatric HIV acquisitions according to maternal HIV status, ART engagement and timing of maternal HIV acquisition during pregnancy or breastfeeding. Transmission pathway estimates were visualized using stacked bar charts (File S1 and Figure 1). Selected country‐level analyses further illustrated heterogeneity in vertical transmission patterns across epidemic settings (Figure 2).

Findings from the decomposition analysis of new HIV acqusitions in childreninformed the need for intervention modelling to evaluate the epidemiological impact and cost‐effectiveness of LAI lenacapavir PrEP. Individual‐level risk‐based targeting approaches were not modelled because harmonized behavioural and clinical risk data were not consistently available [27]. Geographic incidence‐based targeting was, therefore, used as a scalable proxy for programme prioritization.

The analysis followed a longitudinal cohort of HIV‐negative PBW over 2.2 years, representing the interval from the first antenatal care (ANC) visit through median breastfeeding duration [28].

The base‐case scenario reflected operationally plausible implementation within ANC and postnatal care (PNC) systems [29, 30, 31, 32, 33], assuming:

- 65% uptake among eligible HIV‐negative PBW
- 70% retention over 2.2 years, defined as continued attendance for scheduled LAI PrEP administration within ANC/PNC services
- Drug costs based on the projected generic‐access target price of US$40 per person‐year plus a US$17 loading dose, with an additional 20% adjustment for procurement uncertainty, supply chain variation and wastage
- Service delivery costs of US$50 per person‐year derived from oral PrEP micro‐costing studies within maternal and reproductive health services

Uptake and retention reflected continued engagement with scheduled injectable delivery rather than adherence‐based behaviour, which is less applicable to LAI formulations [34].

The upper‐bound scenario assumed:

- 100% uptake and retention over the full protection period
- identical cost structure to the base‐case scenario
- no loss to follow‐up

This scenario represented a theoretical upper bound under full programme coverage and sustained engagement.

Both implementation scenarios were evaluated under:

- Universal rollout across all HIV‐negative PBW aged 15–49 years in SSA, with population estimates derived from Spectrum AIM
- Geographic targeting strategies using subnational HIV incidence thresholds among women aged 15–49 years derived from the Naomi model

Geographic targeting tiers included:

- ≥0.7% incidence (high‐priority targeting)
- ≥0.5% incidence (intermediate targeting)
- ≥0.3% incidence (expanded targeting)

Thresholds were selected a priori to represent plausible programme prioritization strategies rather than cost‐effectiveness cut‐offs.

Estimated outcomes included:

- Number of PBW without HIV reached
- Maternal HIV acquisitions averted
- Paediatric HIV acquisitions averted
- Total HIV acquisitions averted
- Total programme costs

Cost‐effectiveness outcomes included:

- Cost per HIV acquisition averted
- Net cost per HIV acquisition averted, including lifetime ART costs averted
- Net programme costs

Lifetime ART costs were estimated at US$6000 per adult and US$8000 per infant [15].

Deterministic sensitivity analyses used net cost per HIV acquisition averted as the primary outcome across implementation scenarios and geographic targeting strategies.

One‐way sensitivity analyses varied:

- Uptake (50%, 70%, 90%)
- Retention (50%, 70%, 90%)
- Service delivery costs (US$35, US$50, US$75)

Drug procurement costs were not varied because negotiated lenacapavir pricing agreements were assumed to remain relatively stable [29, 30, 31, 32, 33, 35].

All scenarios were evaluated using a consistent epidemiological and costing framework; differences, therefore, reflected implementation assumptions and eligibility thresholds rather than structural model differences.

Multi‐way sensitivity analyses jointly varied uptake, retention and service delivery costs to assess robustness under less favourable implementation conditions.

All analyses and visualizations were conducted in R version 4.4.1 (R Foundation for Statistical Computing, Vienna, Austria).

Ethical approval was not required because the study used publicly available aggregated secondary data.

## 3 Results

In 2024, approximately 40.2 million births occurred in SSA, of which 1.1 million (2.5%) were to women living with HIV. Although 86% of PBW living with HIV received ART to prevent vertical transmission, gaps in coverage, retention and incident maternal HIV acquisition resulted in an estimated 98,000 new paediatric HIV acquisitions, representing 83% of the global burden. These acquisitions resulted from multiple transmission pathways spanning maternal HIV status prior to pregnancy and new HIV acquisition during pregnancy or breastfeeding.

Across SSA, the largest contributor to paediatric HIV acquisitions was insufficient ART access among women living with HIV, accounting for approximately 45,000 acquisitions (46%), predominantly in Western and Central Africa. ART discontinuation during pregnancy or breastfeeding contributed a further 19,000 acquisitions (19%), with a higher burden in Eastern and Southern Africa. Incident maternal HIV acquisition during pregnancy or breastfeeding accounted for approximately 101,000 maternal acquisitions, resulting in 24,100 paediatric acquisitions (25% of all paediatric HIV acquisitions in SSA). This pathway represented a substantial contribution to vertical transmission alongside ART‐related gaps (Figure 1).

Marked heterogeneity was observed across settings. The proportion of vertical transmission attributable to maternal HIV acquisition during pregnancy or breastfeeding was 32% in Eastern and Southern Africa and 15% in Western and Central Africa (Figure 1). At the country level, this pathway accounted for 59% of vertical transmissions in South Africa, 46% in Zambia, 17% in Zimbabwe and 14% in Burkina Faso, reflecting variation in underlying HIV incidence and maternal ART outcomes (Figure 2).

Provision of LAI lenacapavir PrEP to PBW without HIV was evaluated under two implementation scenarios (base‐case and upper‐bound) and four targeting strategies (universal rollout and three geographic incidence thresholds). Under universal rollout, defined as all PBW without HIV aged 15–49 years, the base‐case scenario (65% uptake and 70% retention) reached 25.5 million PBW, resulting in 38.9 million effective person‐years of protection and 56,100 HIV acquisitions averted (44,500 maternal and 11,600 paediatric) over 2.2 years (Table S2A). The upper‐bound scenario (100% uptake and retention) reached 39.2 million PBW, generating 85.6 million person‐years of protection and averting 123,000 HIV acquisitions (97,700 maternal and 25,500 paediatric).

Geographically targeted strategies, based on subnational HIV incidence thresholds among women aged 15–49 years, substantially reduced the number of women reached while preserving a large proportion of the overall impact (Table S2A). At the highest‐priority threshold (≥0.7% incidence), which primarily included districts in South Africa, Mozambique, Zambia and Eswatini, the base‐case scenario reached approximately 626,000 PBW and averted 8450 HIV acquisitions (6700 maternal and 1750 paediatric). Expanding eligibility to districts with incidence ≥0.5% increased coverage to 1.08 million PBW and resulted in 10,400 acquisitions averted under the base‐case scenario. Further expansion to districts with incidence ≥0.3% reached 2 million PBW and averted 11,600 HIV acquisitions. Results under the upper‐bound scenario followed a similar pattern, with approximately twofold greater impact across targeting strategies (Table S2A). Complete model outputs across implementation and targeting strategies are provided in File S2.

Corresponding programme costs and cost‐effectiveness estimates are presented in Table S2B. Under universal rollout, total programme costs were estimated at US$5.14 billion in the base‐case scenario and US$9.18 billion in the upper‐bound scenario, with net costs per HIV acquisition averted of US$85,200 and US$68,100, respectively, after accounting for lifetime ART savings. Targeted strategies were substantially more cost‐efficient. At the ≥0.7% incidence threshold, total programme costs were US$126 million (base‐case) and US$226 million (upper‐bound), yielding the lowest net cost per HIV acquisition averted (US$8530 and US$5750, respectively). Both total costs and net cost per HIV acquisition averted increased with broader eligibility thresholds (≥0.5% and ≥0.3%), although all targeted strategies remained substantially more efficient than universal rollout (Table S2B). Across all scenarios, maternal HIV acquisitions accounted for the majority of HIV acquisitions averted.

**FIGURE 3:**
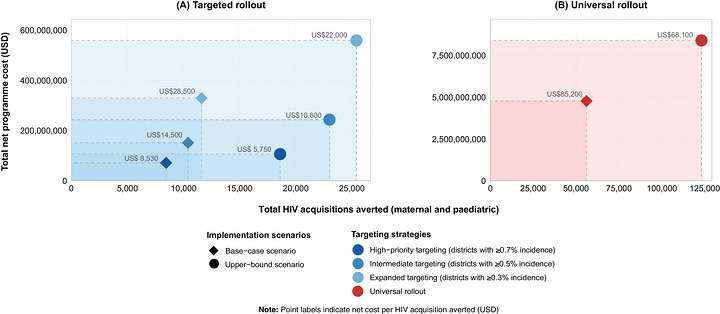
Impact and cost‐effectiveness of universal and targeted lenacapavir pre‐exposure prophylaxis rollout among pregnant and breastfeedingin sub‐Saharan Africa. Panel A shows targeted rollout; Panel B shows universal rollout. Different axis scales are used between panels. The x‐axis represents total HIV acquisitions averted (maternal and paediatric), and the y‐axis represents total net programme costs over 2.2 years (million USD). Connected points illustrate changes in epidemiological impact and net programme costs across geographic targeting strategies.

Overall, expanding geographic eligibility increased the total number of HIV acquisitions averted but was associated with progressively higher programme costs and lower efficiency per HIV acquisition averted. The most selective targeting strategy (≥0.7% incidence) consistently yielded the lowest net cost per HIV acquisition averted, whereas universal rollout achieved the greatest overall impact. Full results are presented in File S2 (Table S2A,B) and visualized in Figure 3.

Deterministic sensitivity analyses across all geographic targeting scenarios showed that net cost per HIV acquisition averted was highly sensitive to retention and service delivery costs but showed minimal sensitivity to uptake. In one‐way sensitivity analyses, variation in uptake (50%–90%) had no meaningful impact on cost‐effectiveness across any targeting strategy, reflecting proportional scaling of programme costs and HIV acquisitions averted under constant per‐person costs and risk assumptions. In contrast, retention was the primary driver of variation. For high‐priority targeting (≥0.7% incidence), net cost per HIV acquisition averted ranged from US$6470 (90% retention) to US$12,200 (50% retention). Under universal rollout, values ranged from US$72,600 to US$108,000. Service delivery costs produced a smaller but consistent gradient, with higher costs leading to worse cost‐effectiveness across all scenarios (File S3).

**FIGURE 4:**
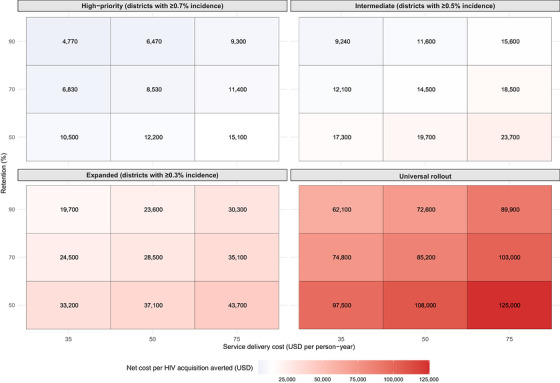
Multi‐way sensitivity of net cost per HIV acquisition averted across retention and service delivery costs under the base‐case implementation scenario, by geographic targeting strategy. Heat maps show net cost per HIV acquisition averted across combinations of retention (y‐axis) and service delivery cost (x‐axis) under the base‐case implementation scenario. Panels represent geographic targeting strategies. Values are derived from multi‐way sensitivity analysis across parameter combinations.

Multi‐way sensitivity analysis across the 27 combinations of uptake, retention and service delivery costs confirmed these patterns (File S4 and Figure 4). Retention remained the dominant determinant of cost‐effectiveness across all geographic targeting strategies. For high‐priority targeting (≥0.7% incidence), net cost per HIV acquisition averted ranged from US$4770 under the most favourable assumptions (90% uptake, 90% retention, US$35 service delivery cost) to US$15,100 under the least favourable assumptions (50% uptake, 50% retention, US$75 service delivery cost). Universal rollout ranged from US$62,100 to US$125,000 across the same parameter space. Importantly, these patterns were consistent across both base‐case and upper‐bound implementation scenarios, with variation in absolute values but no change in ranking or key drivers of cost‐effectiveness. Across all scenarios, incidence‐based geographic targeting remained more economically efficient than universal rollout, including under pessimistic assumptions. Overall, retention in care emerged as the primary implementation determinant of cost‐effectiveness, with service delivery costs playing a secondary role and uptake having an insignificant influence.

## 4 Discussion

Despite major progress in reducing paediatric HIV acquisitions across SSA, substantial gaps remain across the maternal HIV prevention and treatment cascade. Our decomposition analysis showed that insufficient maternal ART access remains the largest contributor to vertical HIV transmission in SSA (46%), followed by maternal HIV acquisition during pregnancy or breastfeeding (25%) and ART discontinuation during pregnancy or breastfeeding (19%) (Figure 1). The relative contribution of maternal HIV acquisition varied substantially across settings, accounting for 59% of vertical transmission in South Africa and 46% in Zambia, highlighting important epidemiological heterogeneity and the need for context‐specific prevention strategies (Figure 2).

These findings are consistent with empirical evidence showing that vertical transmission risk remains strongly influenced by gaps in ART initiation, retention and viral suppression among PBW. For example, the Burkina Faso PROMISE‐EPI study demonstrated that even among women aware of their HIV status and receiving ART, suboptimal viral suppression associated with poor adherence and retention substantially increased transmission risk [36, 37, 38, 39, 40, 41]. Given that the probability of vertical HIV transmission without ART ranges from 30% to 45%, even modest disruptions across the prevention cascade can result in substantial increases in paediatric HIV acquisitions [5, 23].

Twice‐yearly LAI lenacapavir PrEP represents a potentially important complementary prevention strategy for reducing maternal HIV acquisition during pregnancy and breastfeeding [17, 18, 19]. Our modelling suggests that geographically targeted implementation in high‐incidence districts is substantially more economically efficient than universal rollout across SSA. The most selective targeting strategy (≥0.7% incidence) consistently achieved the lowest net cost per acquisition averted across all implementation scenarios and sensitivity analyses. However, once base‐case implementation assumptions were incorporated, including service delivery costs and imperfect retention, net costs increased substantially across all strategies (Figure 4). These findings are consistent with recent modelling from Eastern and Southern Africa suggesting that long‐acting PrEP may require substantially lower fully loaded implementation costs to meet conventional cost‐effectiveness thresholds [30, 31].

Although daily oral PrEP remains the current standard prevention approach, its effectiveness among PBW is frequently constrained by low adherence and high discontinuation rates during pregnancy and the postpartum period [8, 9, 42]. Preference studies suggest that many women favour LAI options because of reduced pill burden, greater discretion and less frequent dosing requirements [43, 44]. However, the successful implementation of LAI lenacapavir PrEP will require more than drug availability alone. Integration into routine antenatal, postnatal and maternal−child health services, alongside demand generation, community engagement and retention support, will be critical to achieving meaningful population‐level impact. Persistent structural barriers, including stigma, socioeconomic inequities and limitations in healthcare access, continue to shape HIV vulnerability and programme engagement across many settings [10, 11, 12, 13].

Additional modelling from South Africa using the Thembisa model (version 4.8) similarly suggests that increasing PrEP uptake among women at elevated HIV risk through the introduction of lenacapavir could substantially reduce new HIV acquisitions, including among PBW [34, 35]. Together, these findings support the potential role of LAI PrEP as part of a broader combination prevention strategy in high‐incidence settings.

Importantly, LAI lenacapavir PrEP should be viewed as complementary to, rather than a substitute for, strengthening existing vertical transmission programmes. Optimizing HIV testing, ART initiation, retention in care and viral suppression among women living with HIV remains the single most important priority for reducing paediatric HIV acquisitions in SSA, as these gaps continue to account for the majority of vertical transmission burden. Geographic prioritization of LAI PrEP may nevertheless provide an important secondary prevention strategy when efficiently integrated alongside existing maternal HIV services.

This analysis evaluated both plausible (base‐case) and optimistic implementation (upper‐bound) scenarios and incorporated deterministic one‐way and multi‐way sensitivity analyses to examine the robustness of epidemiological and economic outcomes under varying assumptions regarding uptake, retention and service delivery costs. However, several limitations should be considered.

First, service delivery costs were estimated using oral PrEP micro‐costing studies as proxy estimates [29, 31, 32], which may not fully capture the operational requirements associated with LAI PrEP delivery, including cold‐chain logistics, workforce training, pharmacovigilance and repeated clinic‐based administration. Actual implementation costs may, therefore, differ substantially across settings.

Second, although the model assumed a 2.2‐year protection period spanning pregnancy through the median breastfeeding duration in SSA [28], real‐world retention in HIV prevention services frequently declines during the postpartum period. Lower retention following delivery would likely reduce total acquisitions averted and worsen cost‐effectiveness outcomes.

Third, the analysis focused exclusively on geographic incidence‐based targeting strategies because harmonized population‐level behavioural and clinical risk data were not consistently available across SSA settings. Individual‐level risk‐based prioritization approaches may improve programme efficiency in some contexts and should be evaluated in future analyses.

Finally, the modelling framework relied on projected implementation assumptions because large‐scale real‐world deployment data for twice‐yearly lenacapavir PrEP among PBW are not yet available. Future implementation research incorporating real‐world uptake, discontinuation, user preferences and integrated maternal healthcare delivery costs will be important for refining estimates of programme effectiveness and long‐term sustainability.

## 5 Conclusions

Despite major progress in reducing paediatric HIV acqusitions in SSA, elimination of vertical HIV transmission will require continued strengthening of maternal HIV testing, ART initiation, retention in care and viral suppression among PBW living with HIV. Maternal HIV acquisition during pregnancy or breastfeeding now accounts for approximately one‐quarter of new paediatric HIV acquisitions across SSA, representing an important residual transmission pathway.

Targeted deployment of twice‐yearly LAI lenacapavir PrEP among PBW without HIV in high‐incidence districts could substantially reduce maternal and paediatric HIV acquisitions. However, under current implementation assumptions and projected costs, universal rollout is unlikely to be economically efficient. Geographic prioritization strategies substantially improve economic efficiency but remain sensitive to implementation costs and retention in care. LAI lenacapavir PrEP should, therefore, be considered a complementary component of broader vertical HIV prevention strategies rather than a replacement for strengthening core maternal HIV services. Integration within routine ANC/PNC systems, alongside efforts to address structural barriers to engagement and retention, will be essential to maximize population‐level impact [45].

## Author Contributions

MM, JS, LFJ and AY conceived the study and developed the overall research framework. AY led the manuscript development and writing, conducted the primary analyses and sensitivity analyses, cost‐effectiveness and sensitivity analyses, developed the implementation and geographic targeting scenarios, designed all figures and visualizations, prepared the Supplementary Materials, and coordinated revisions in response to reviewer and editorial feedback. MM, JS, AY, MKW, and DM contributed to the conceptualization of the modelling framework, including universal rollout and geographically targeted implementation strategies informed by Naomi's subnational incidence estimates. LFJ contributed to the development of the risk‐profiling framework for women at elevated risk of HIV acquisition in South Africa. AM provided country‐specific programmatic and implementation expertise. JS, DM, GMR, and LJ contributed to the cost estimation and cost‐effectiveness analysis framework, with AY leading the analytical implementation. SB provided technical insights on maternal and child health from a UNICEF perspective. MM provided overall scientific oversight and ensured alignment with global HIV policy frameworks and guidelines. All authors contributed to the interpretation of findings, critically revised the manuscript for important intellectual content and approved the final version for submission.

## Funding

This research did not receive any specific grants from funding agencies in the public, commercial or not‐for‐profit sectors. MKW received funding from the National Institute of Allergy and Infectious Diseases of the National Institutes of Health under award number 1R01AI152721‐01A1, and from the UK Medical Research Council (MRC) Centre for Global Infectious Disease Analysis (reference MR/R015600/1). This funding was jointly provided by the MRC and the UK Foreign, Commonwealth & Development Office (FCDO) under the MRC/FCDO Concordat agreement and as part of the EDCTP2 programme supported by the European Union.

## Conflicts of Interest

The authors declare no conflicts of interest.

## Supporting information




**File S1**: Decomposition of paediatric HIV acquisitions by timing of maternal HIV acquisition and antiretroviral therapy status (Spectrum AIDS Impact Module stacked bar analysis).

**File S2**: Full model results across implementation scenarios and targeting strategies for lenacapavir pre‐exposure prophylaxis rollout in sub‐Saharan Africa.

**File S3**: One‐way sensitivity analysis of net costs per HIV acquisition averted under base‐case implementation assumptions across geographic targeting strategies for lenacapavir pre‐exposure prophylaxis rollout in sub‐Saharan Africa.

**File S4**: Multi‐way sensitivity analysis of net costs per HIV acquisition averted under base‐case implementation assumptions across geographic targeting strategies for lenacapavir pre‐exposure prophylaxis rollout in sub‐Saharan Africa.

## Data Availability

The epidemiological estimates used in this analysis are publicly accessible via UNAIDS at https://aidsinfo.unaids.org. All additional data supporting these findings can be requested from the corresponding author.
