# Supplementary material for: Drivers of Vertical HIV Transmission in Sub‐Saharan Africa and the Impact and Cost‐Effectiveness of Targeted and Universal Lenacapavir Pre‐Exposure Prophylaxis
Source: J Int AIDS Soc. 2026 Jun 19;29(Suppl 1):e70127. doi: 10.1002/jia2.70127 (PMC13281411; doi:10.1002/jia2.70127)
Supplement: Supplementary file 1 — File S1: Decomposition of paediatric HIV acquisitions by timing of maternal HIV acquisition and antiretroviral therapy status (Spectrum AIDS Impact Module stacked bar analysis). [file JIA2-29-e70127-s004.docx]

**Supporting Information File S1: Decomposition of paediatric HIV acquisitions by timing of maternal HIV acquisition and antiretroviral therapy status (Spectrum AIM stacked bar analysis)**

| Timing of maternal HIV acquisition / transmission* | Maternal ART status | Timing of vertical transmission |
| --- | --- | --- |
| Mother infected during pregnancy | Women not receiving ART who acquired HIV during pregnancy | By 6-8 weeks after birth |
| Did not receive antiretroviral therapy during pregnancy | Not receiving ART |  |
| Dropped off antiretroviral therapy during pregnancy; child infected during pregnancy | Treatment started before pregnancy, discontinued before delivery |  |
| Started antiretroviral therapy late in the pregnancy; child infected during pregnancy | Treatment started less than four weeks before delivery (in ANC), with suboptimal outcomes |  |
| Started antiretroviral therapy during the pregnancy; child infected during pregnancy | Treatment started in ANC, with suboptimal outcomes |  |
| Started antiretroviral therapy before the pregnancy; child infected during pregnancy | Treatment started before pregnancy, with suboptimal outcomes |  |
| Mother infected during breastfeeding | Women not receiving ART who acquired HIV during breastfeeding | During breastfeeding |
| Did not receive antiretroviral therapy during breastfeeding | Not receiving ART |  |
| Dropped off antiretroviral therapy; child infected during breastfeeding | Treatment started before pregnancy, discontinued during pregnancy or breastfeeding |  |
| Started antiretroviral therapy late in pregnancy; child infected during breastfeeding | Treatment started less than four weeks before delivery (in ANC), with suboptimal outcomes |  |
| Started antiretroviral therapy during pregnancy; child infected during breastfeeding | Treatment started during ANC, with suboptimal outcomes |  |
| Started antiretroviral therapy before pregnancy; child infected during breastfeeding | Treatment started before pregnancy, with suboptimal outcomes |  |

*Note: The first column reproduces Spectrum AIM stacked bar analysis output categories verbatim to preserve traceability to model outputs. Interpretive descriptions in the remaining columns use people-first terminology.
