# Supplementary material for: Drivers of Vertical HIV Transmission in Sub‐Saharan Africa and the Impact and Cost‐Effectiveness of Targeted and Universal Lenacapavir Pre‐Exposure Prophylaxis
Source: J Int AIDS Soc. 2026 Jun 19;29(Suppl 1):e70127. doi: 10.1002/jia2.70127 (PMC13281411; doi:10.1002/jia2.70127)
Supplement: Supplementary file 2 — File S2: Full model results across implementation scenarios and targeting strategies for lenacapavir pre‐exposure prophylaxis rollout in sub‐Saharan Africa. [file JIA2-29-e70127-s002.docx]

**Supporting Information File S2: Full model results across implementation scenarios and targeting strategies for lenacapavir pre-exposure prophylaxis rollout in sub-Saharan Africa**

**Table S2A. Epidemiological impact and coverage outcomes**

| Implementation scenario | Targeting strategy | Pregnant and breastfeeding women reached | Effective person-years protected | Maternal HIV acquisitions averted | Paediatric HIV acquisitions averted | Total HIV acquisitions averted |
| --- | --- | --- | --- | --- | --- | --- |
| Base−case scenario | Universal rollout | 25,500,000 | 38,925,800 | 44,500 | 11,600 | 56,100 |
| Upper−bound scenario | Universal rollout | 39,200,000 | 85,551,209 | 97,700 | 25,500 | 123,000 |
| Base−case scenario | ≥0.3% | 2,000,000 | 3,059,067 | 9,180 | 2,390 | 11,600 |
| Upper−bound scenario | ≥0.3% | 3,080,000 | 6,723,223 | 20,200 | 5,260 | 25,400 |
| Base−case scenario | ≥0.5% | 1,080,000 | 1,656,877 | 8,280 | 2,160 | 10,400 |
| Upper−bound scenario | ≥0.5% | 1,670,000 | 3,641,488 | 18,200 | 4,750 | 23,000 |
| Base−case scenario | ≥0.7% | 626,000 | 957,333 | 6,700 | 1,750 | 8,450 |
| Upper−bound scenario | ≥0.7% | 964,000 | 2,104,028 | 14,700 | 3,840 | 18,600 |

**Table S2B. Economic and cost-effectiveness outcomes**

| Implementation scenario | Targeting strategy | Drug cost (USD) | Service delivery cost (USD) | Total programme cost (USD) | Lifetime ART savings (USD) | Net programme cost (USD) | Cost per acquisition averted (USD) | Net cost per acquisition averted (USD) |
| --- | --- | --- | --- | --- | --- | --- | --- | --- |
| Base−case scenario | Universal rollout | 3,188,800,000 | 1,946,300,000 | 5,135,100,000 | 359,500,000 | 4,775,500,000 | 91,600 | 85,200 |
| Upper−bound scenario | Universal rollout | 4,905,800,000 | 4,277,600,000 | 9,183,400,000 | 790,200,000 | 8,393,200,000 | 74,500 | 68,100 |
| Base−case scenario | ≥0.3% | 250,600,000 | 153,000,000 | 403,500,000 | 74,200,000 | 329,300,000 | 34,900 | 28,500 |
| Upper−bound scenario | ≥0.3% | 385,500,000 | 336,200,000 | 721,700,000 | 163,100,000 | 558,600,000 | 28,400 | 22,000 |
| Base−case scenario | ≥0.5% | 135,700,000 | 82,800,000 | 218,600,000 | 67,000,000 | 151,600,000 | 20,900 | 14,500 |
| Upper−bound scenario | ≥0.5% | 208,800,000 | 182,100,000 | 390,900,000 | 147,200,000 | 243,600,000 | 17,000 | 10,600 |
| Base−case scenario | ≥0.7% | 78,400,000 | 47,900,000 | 126,300,000 | 54,200,000 | 72,100,000 | 14,900 | 8,530 |
| Upper−bound scenario | ≥0.7% | 120,700,000 | 105,200,000 | 225,900,000 | 119,100,000 | 106,700,000 | 12,200 | 5,750 |

Targeting thresholds represent districts with HIV incidence among women aged 15-49 years of ≥0.7%, ≥0.5%, and ≥0.3%; universal rollout represents all pregnant and breastfeeding women without HIV aged 15-49 years in sub-Saharan Africa irrespective of district-level incidence.

Universal rollout based on UNAIDS epidemiological estimates (2025). The total eligible population comprised 39.2 million pregnant and breastfeeding women without HIV across 45 countries in sub-Saharan Africa, with 16.36 million in Eastern and Southern Africa and 22.82 million in Western and Central Africa.

Targeted rollout based on subnational HIV incidence thresholds among women aged 15–49 years (derived from the Naomi model):

- High-priority targeting (districts with ≥0.7% incidence): Approximately 0.96 million pregnant and breastfeeding women without HIV were eligible, distributed as follows: Eswatini (n=22,000), Mozambique (n=461,000), South Africa (n=470,000), Zambia (n=10,000).
- Intermediate targeting (districts with ≥0.5% incidence): Approximately 1.67 million pregnant and breastfeeding women without HIV were eligible across nine countries: Botswana (n=12,000), Congo (n=39,000), Eswatini (n=22,000), Gabon (n=1,000), Mozambique (n=664,000), Namibia (n=26,000), South Africa (n=744,000), Uganda (n=5,000), Zambia (n=154,000).
- Expanded targeting (districts with ≥0.3% incidence): Approximately 3.08 million pregnant and breastfeeding women without HIV were eligible across 18 countries in sub-Saharan Africa.
