# Supplementary material for: Drivers of Vertical HIV Transmission in Sub‐Saharan Africa and the Impact and Cost‐Effectiveness of Targeted and Universal Lenacapavir Pre‐Exposure Prophylaxis
Source: J Int AIDS Soc. 2026 Jun 19;29(Suppl 1):e70127. doi: 10.1002/jia2.70127 (PMC13281411; doi:10.1002/jia2.70127)
Supplement: Supplementary file 3 — File S3: One‐way sensitivity analysis of net costs per HIV acquisition averted under base‐case implementation assumptions across geographic targeting strategies for lenacapavir pre‐exposure prophylaxis rollout in sub‐Saharan Africa. [file JIA2-29-e70127-s001.docx]

**Supporting Information File S3: One-way sensitivity analysis of net costs per HIV acquisition averted under base-case implementation assumptions across geographic targeting strategies for lenacapavir pre-exposure prophylaxis rollout in sub-Saharan Africa**

| Targeting strategy | Parameter varied | Tested value | Uptake (%) | Retention (%) | Service cost (USD per person-year) | Net cost per HIV acquisition averted (USD) |
| --- | --- | --- | --- | --- | --- | --- |
| ≥0.7% | Uptake (%) | 50 | 50 | 70 | 50 | 8,530 |
| ≥0.7% | Uptake (%) | 70 | 70 | 70 | 50 | 8,530 |
| ≥0.7% | Uptake (%) | 90 | 90 | 70 | 50 | 8,530 |
| ≥0.7% | Retention (%) | 50 | 65 | 50 | 50 | 12,200 |
| ≥0.7% | Retention (%) | 70 | 65 | 70 | 50 | 8,530 |
| ≥0.7% | Retention (%) | 90 | 65 | 90 | 50 | 6,470 |
| ≥0.7% | Service delivery cost (USD per person-year) | 35 | 65 | 70 | 35 | 6,830 |
| ≥0.7% | Service delivery cost (USD per person-year) | 50 | 65 | 70 | 50 | 8,530 |
| ≥0.7% | Service delivery cost (USD per person-year) | 75 | 65 | 70 | 75 | 11,400 |
| ≥0.5% | Uptake (%) | 50 | 50 | 70 | 50 | 14,500 |
| ≥0.5% | Uptake (%) | 70 | 70 | 70 | 50 | 14,500 |
| ≥0.5% | Uptake (%) | 90 | 90 | 70 | 50 | 14,500 |
| ≥0.5% | Retention (%) | 50 | 65 | 50 | 50 | 19,700 |
| ≥0.5% | Retention (%) | 70 | 65 | 70 | 50 | 14,500 |
| ≥0.5% | Retention (%) | 90 | 65 | 90 | 50 | 11,600 |
| ≥0.5% | Service delivery cost (USD per person-year) | 35 | 65 | 70 | 35 | 12,100 |
| ≥0.5% | Service delivery cost (USD per person-year) | 50 | 65 | 70 | 50 | 14,500 |
| ≥0.5% | Service delivery cost (USD per person-year) | 75 | 65 | 70 | 75 | 18,500 |
| ≥0.3% | Uptake (%) | 50 | 50 | 70 | 50 | 28,500 |
| ≥0.3% | Uptake (%) | 70 | 70 | 70 | 50 | 28,500 |
| ≥0.3% | Uptake (%) | 90 | 90 | 70 | 50 | 28,500 |
| ≥0.3% | Retention (%) | 50 | 65 | 50 | 50 | 37,100 |
| ≥0.3% | Retention (%) | 70 | 65 | 70 | 50 | 28,500 |
| ≥0.3% | Retention (%) | 90 | 65 | 90 | 50 | 23,600 |
| ≥0.3% | Service delivery cost (USD per person-year) | 35 | 65 | 70 | 35 | 24,500 |
| ≥0.3% | Service delivery cost (USD per person-year) | 50 | 65 | 70 | 50 | 28,500 |
| ≥0.3% | Service delivery cost (USD per person-year) | 75 | 65 | 70 | 75 | 35,100 |
| Universal rollout | Uptake (%) | 50 | 50 | 70 | 50 | 85,200 |
| Universal rollout | Uptake (%) | 70 | 70 | 70 | 50 | 85,200 |
| Universal rollout | Uptake (%) | 90 | 90 | 70 | 50 | 85,200 |
| Universal rollout | Retention (%) | 50 | 65 | 50 | 50 | 108,000 |
| Universal rollout | Retention (%) | 70 | 65 | 70 | 50 | 85,200 |
| Universal rollout | Retention (%) | 90 | 65 | 90 | 50 | 72,600 |
| Universal rollout | Service delivery cost (USD per person-year) | 35 | 65 | 70 | 35 | 74,800 |
| Universal rollout | Service delivery cost (USD per person-year) | 50 | 65 | 70 | 50 | 85,200 |
| Universal rollout | Service delivery cost (USD per person-year) | 75 | 65 | 70 | 75 | 103,000 |

Targeting thresholds represent districts with HIV incidence among women aged 15-49 years of ≥0.7%, ≥0.5%, and ≥0.3%; universal rollout represents all pregnant and breastfeeding women without HIV aged 15-49 years in sub-Saharan Africa irrespective of district-level incidence.
