# Supplementary material for: Drivers of Vertical HIV Transmission in Sub‐Saharan Africa and the Impact and Cost‐Effectiveness of Targeted and Universal Lenacapavir Pre‐Exposure Prophylaxis
Source: J Int AIDS Soc. 2026 Jun 19;29(Suppl 1):e70127. doi: 10.1002/jia2.70127 (PMC13281411; doi:10.1002/jia2.70127)
Supplement: Supplementary file 4 — File S4: Multi‐way sensitivity analysis of net costs per HIV acquisition averted under base‐case implementation assumptions across geographic targeting strategies for lenacapavir pre‐exposure prophylaxis rollout in sub‐Saharan Africa. [file JIA2-29-e70127-s003.docx]

**Supporting Information File S4: Multi-way sensitivity analysis of net costs per HIV acquisition averted under base-case implementation assumptions across geographic targeting strategies for lenacapavir pre-exposure prophylaxis rollout in sub-Saharan Africa**

| Targeting strategy | Uptake rate  (%) | Retention rate (%) | Service delivery cost (USD per person-year) | Net cost per HIV acquisition averted (USD) | Maternal HIV acquisition averted | Paediatric HIV acquisition averted | Total HIV acquisition averted |
| --- | --- | --- | --- | --- | --- | --- | --- |
| ≥0.7% | 50 | 50 | 35 | 10,500 | 3,680 | 961 | 4,640 |
| ≥0.7% | 50 | 50 | 50 | 12,200 | 3,680 | 961 | 4,640 |
| ≥0.7% | 50 | 50 | 75 | 15,100 | 3,680 | 961 | 4,640 |
| ≥0.7% | 50 | 70 | 35 | 6,830 | 5,160 | 1,340 | 6,500 |
| ≥0.7% | 50 | 70 | 50 | 8,530 | 5,160 | 1,340 | 6,500 |
| ≥0.7% | 50 | 70 | 75 | 11,400 | 5,160 | 1,340 | 6,500 |
| ≥0.7% | 50 | 90 | 35 | 4,770 | 6,630 | 1,730 | 8,360 |
| ≥0.7% | 50 | 90 | 50 | 6,470 | 6,630 | 1,730 | 8,360 |
| ≥0.7% | 50 | 90 | 75 | 9,300 | 6,630 | 1,730 | 8,360 |
| ≥0.7% | 70 | 50 | 35 | 10,500 | 5,160 | 1,340 | 6,500 |
| ≥0.7% | 70 | 50 | 50 | 12,200 | 5,160 | 1,340 | 6,500 |
| ≥0.7% | 70 | 50 | 75 | 15,100 | 5,160 | 1,340 | 6,500 |
| ≥0.7% | 70 | 70 | 35 | 6,830 | 7,220 | 1,880 | 9,100 |
| ≥0.7% | 70 | 70 | 50 | 8,530 | 7,220 | 1,880 | 9,100 |
| ≥0.7% | 70 | 70 | 75 | 11,400 | 7,220 | 1,880 | 9,100 |
| ≥0.7% | 70 | 90 | 35 | 4,770 | 9,280 | 2,420 | 11,700 |
| ≥0.7% | 70 | 90 | 50 | 6,470 | 9,280 | 2,420 | 11,700 |
| ≥0.7% | 70 | 90 | 75 | 9,300 | 9,280 | 2,420 | 11,700 |
| ≥0.7% | 90 | 50 | 35 | 10,500 | 6,630 | 1,730 | 8,360 |
| ≥0.7% | 90 | 50 | 50 | 12,200 | 6,630 | 1,730 | 8,360 |
| ≥0.7% | 90 | 50 | 75 | 15,100 | 6,630 | 1,730 | 8,360 |
| ≥0.7% | 90 | 70 | 35 | 6,830 | 9,280 | 2,420 | 11,700 |
| ≥0.7% | 90 | 70 | 50 | 8,530 | 9,280 | 2,420 | 11,700 |
| ≥0.7% | 90 | 70 | 75 | 11,400 | 9,280 | 2,420 | 11,700 |
| ≥0.7% | 90 | 90 | 35 | 4,770 | 11,900 | 3,110 | 15,000 |
| ≥0.7% | 90 | 90 | 50 | 6,470 | 11,900 | 3,110 | 15,000 |
| ≥0.7% | 90 | 90 | 75 | 9,300 | 11,900 | 3,110 | 15,000 |
| ≥0.5% | 50 | 50 | 35 | 17,300 | 4,550 | 1,190 | 5,740 |
| ≥0.5% | 50 | 50 | 50 | 19,700 | 4,550 | 1,190 | 5,740 |
| ≥0.5% | 50 | 50 | 75 | 23,700 | 4,550 | 1,190 | 5,740 |
| ≥0.5% | 50 | 70 | 35 | 12,100 | 6,370 | 1,660 | 8,040 |
| ≥0.5% | 50 | 70 | 50 | 14,500 | 6,370 | 1,660 | 8,040 |
| ≥0.5% | 50 | 70 | 75 | 18,500 | 6,370 | 1,660 | 8,040 |
| ≥0.5% | 50 | 90 | 35 | 9,240 | 8,190 | 2,140 | 10,300 |
| ≥0.5% | 50 | 90 | 50 | 11,600 | 8,190 | 2,140 | 10,300 |
| ≥0.5% | 50 | 90 | 75 | 15,600 | 8,190 | 2,140 | 10,300 |
| ≥0.5% | 70 | 50 | 35 | 17,300 | 6,370 | 1,660 | 8,040 |
| ≥0.5% | 70 | 50 | 50 | 19,700 | 6,370 | 1,660 | 8,040 |
| ≥0.5% | 70 | 50 | 75 | 23,700 | 6,370 | 1,660 | 8,040 |
| ≥0.5% | 70 | 70 | 35 | 12,100 | 8,920 | 2,330 | 11,200 |
| ≥0.5% | 70 | 70 | 50 | 14,500 | 8,920 | 2,330 | 11,200 |
| ≥0.5% | 70 | 70 | 75 | 18,500 | 8,920 | 2,330 | 11,200 |
| ≥0.5% | 70 | 90 | 35 | 9,240 | 11,500 | 2,990 | 14,500 |
| ≥0.5% | 70 | 90 | 50 | 11,600 | 11,500 | 2,990 | 14,500 |
| ≥0.5% | 70 | 90 | 75 | 15,600 | 11,500 | 2,990 | 14,500 |
| ≥0.5% | 90 | 50 | 35 | 17,300 | 8,190 | 2,140 | 10,300 |
| ≥0.5% | 90 | 50 | 50 | 19,700 | 8,190 | 2,140 | 10,300 |
| ≥0.5% | 90 | 50 | 75 | 23,700 | 8,190 | 2,140 | 10,300 |
| ≥0.5% | 90 | 70 | 35 | 12,100 | 11,500 | 2,990 | 14,500 |
| ≥0.5% | 90 | 70 | 50 | 14,500 | 11,500 | 2,990 | 14,500 |
| ≥0.5% | 90 | 70 | 75 | 18,500 | 11,500 | 2,990 | 14,500 |
| ≥0.5% | 90 | 90 | 35 | 9,240 | 14,700 | 3,850 | 18,600 |
| ≥0.5% | 90 | 90 | 50 | 11,600 | 14,700 | 3,850 | 18,600 |
| ≥0.5% | 90 | 90 | 75 | 15,600 | 14,700 | 3,850 | 18,600 |
| ≥0.3% | 50 | 50 | 35 | 33,200 | 5,040 | 1,320 | 6,360 |
| ≥0.3% | 50 | 50 | 50 | 37,100 | 5,040 | 1,320 | 6,360 |
| ≥0.3% | 50 | 50 | 75 | 43,700 | 5,040 | 1,320 | 6,360 |
| ≥0.3% | 50 | 70 | 35 | 24,500 | 7,060 | 1,840 | 8,900 |
| ≥0.3% | 50 | 70 | 50 | 28,500 | 7,060 | 1,840 | 8,900 |
| ≥0.3% | 50 | 70 | 75 | 35,100 | 7,060 | 1,840 | 8,900 |
| ≥0.3% | 50 | 90 | 35 | 19,700 | 9,080 | 2,370 | 11,400 |
| ≥0.3% | 50 | 90 | 50 | 23,600 | 9,080 | 2,370 | 11,400 |
| ≥0.3% | 50 | 90 | 75 | 30,300 | 9,080 | 2,370 | 11,400 |
| ≥0.3% | 70 | 50 | 35 | 33,200 | 7,060 | 1,840 | 8,900 |
| ≥0.3% | 70 | 50 | 50 | 37,100 | 7,060 | 1,840 | 8,900 |
| ≥0.3% | 70 | 50 | 75 | 43,700 | 7,060 | 1,840 | 8,900 |
| ≥0.3% | 70 | 70 | 35 | 24,500 | 9,880 | 2,580 | 12,500 |
| ≥0.3% | 70 | 70 | 50 | 28,500 | 9,880 | 2,580 | 12,500 |
| ≥0.3% | 70 | 70 | 75 | 35,100 | 9,880 | 2,580 | 12,500 |
| ≥0.3% | 70 | 90 | 35 | 19,700 | 12,700 | 3,320 | 16,000 |
| ≥0.3% | 70 | 90 | 50 | 23,600 | 12,700 | 3,320 | 16,000 |
| ≥0.3% | 70 | 90 | 75 | 30,300 | 12,700 | 3,320 | 16,000 |
| ≥0.3% | 90 | 50 | 35 | 33,200 | 9,080 | 2,370 | 11,400 |
| ≥0.3% | 90 | 50 | 50 | 37,100 | 9,080 | 2,370 | 11,400 |
| ≥0.3% | 90 | 50 | 75 | 43,700 | 9,080 | 2,370 | 11,400 |
| ≥0.3% | 90 | 70 | 35 | 24,500 | 12,700 | 3,320 | 16,000 |
| ≥0.3% | 90 | 70 | 50 | 28,500 | 12,700 | 3,320 | 16,000 |
| ≥0.3% | 90 | 70 | 75 | 35,100 | 12,700 | 3,320 | 16,000 |
| ≥0.3% | 90 | 90 | 35 | 19,700 | 16,300 | 4,260 | 20,600 |
| ≥0.3% | 90 | 90 | 50 | 23,600 | 16,300 | 4,260 | 20,600 |
| ≥0.3% | 90 | 90 | 75 | 30,300 | 16,300 | 4,260 | 20,600 |
| Universal rollout | 50 | 50 | 35 | 97,500 | 24,400 | 6,370 | 30,800 |
| Universal rollout | 50 | 50 | 50 | 108,000 | 24,400 | 6,370 | 30,800 |
| Universal rollout | 50 | 50 | 75 | 125,000 | 24,400 | 6,370 | 30,800 |
| Universal rollout | 50 | 70 | 35 | 74,800 | 34,200 | 8,920 | 43,100 |
| Universal rollout | 50 | 70 | 50 | 85,200 | 34,200 | 8,920 | 43,100 |
| Universal rollout | 50 | 70 | 75 | 103,000 | 34,200 | 8,920 | 43,100 |
| Universal rollout | 50 | 90 | 35 | 62,100 | 44,000 | 11,500 | 55,400 |
| Universal rollout | 50 | 90 | 50 | 72,600 | 44,000 | 11,500 | 55,400 |
| Universal rollout | 50 | 90 | 75 | 89,900 | 44,000 | 11,500 | 55,400 |
| Universal rollout | 70 | 50 | 35 | 97,500 | 34,200 | 8,920 | 43,100 |
| Universal rollout | 70 | 50 | 50 | 108,000 | 34,200 | 8,920 | 43,100 |
| Universal rollout | 70 | 50 | 75 | 125,000 | 34,200 | 8,920 | 43,100 |
| Universal rollout | 70 | 70 | 35 | 74,800 | 47,900 | 12,500 | 60,400 |
| Universal rollout | 70 | 70 | 50 | 85,200 | 47,900 | 12,500 | 60,400 |
| Universal rollout | 70 | 70 | 75 | 103,000 | 47,900 | 12,500 | 60,400 |
| Universal rollout | 70 | 90 | 35 | 62,100 | 61,600 | 16,100 | 77,600 |
| Universal rollout | 70 | 90 | 50 | 72,600 | 61,600 | 16,100 | 77,600 |
| Universal rollout | 70 | 90 | 75 | 89,900 | 61,600 | 16,100 | 77,600 |
| Universal rollout | 90 | 50 | 35 | 97,500 | 44,000 | 11,500 | 55,400 |
| Universal rollout | 90 | 50 | 50 | 108,000 | 44,000 | 11,500 | 55,400 |
| Universal rollout | 90 | 50 | 75 | 125,000 | 44,000 | 11,500 | 55,400 |
| Universal rollout | 90 | 70 | 35 | 74,800 | 61,600 | 16,100 | 77,600 |
| Universal rollout | 90 | 70 | 50 | 85,200 | 61,600 | 16,100 | 77,600 |
| Universal rollout | 90 | 70 | 75 | 103,000 | 61,600 | 16,100 | 77,600 |
| Universal rollout | 90 | 90 | 35 | 62,100 | 79,100 | 20,600 | 99,800 |
| Universal rollout | 90 | 90 | 50 | 72,600 | 79,100 | 20,600 | 99,800 |
| Universal rollout | 90 | 90 | 75 | 89,900 | 79,100 | 20,600 | 99,800 |

Targeting thresholds represent districts with HIV incidence among women aged 15-49 years of ≥0.7%, ≥0.5%, and ≥0.3%; universal rollout represents all pregnant and breastfeeding women without HIV aged 15-49 years in sub-Saharan Africa irrespective of district-level incidence.
